# Occupational exposure to asphalt mixture during road paving is related to increased mitochondria DNA copy number: a cross-sectional study

**DOI:** 10.1186/s12940-018-0375-0

**Published:** 2018-03-27

**Authors:** Yiyi Xu, Christian H. Lindh, Bo A. G. Jönsson, Karin Broberg, Maria Albin

**Affiliations:** 1 Division of Occupational and Environmental Medicine, Laboratory Medicine, Lund University, Scheelevägen 2, 223 63 Lund, Sweden; 2 Unit of Metals & Health, Institute of Environmental Medicine, Karolinska Institutet, Stockholm, Sweden; 3 Unit of Occupational Medicine, Institute of Environmental Medicine, Karolinska Institutet, Stockholm, Sweden

**Keywords:** Mitochondrial DNA, Telomere length, Asphalt, Occupational exposure polyaromatic hydrocarbons

## Abstract

**Background:**

Asphalt workers are exposed to polyaromatic hydrocarbons (PAHs) from hot mix asphalt via both inhalation and dermal absorption. The use of crumb rubber modified (CRM) asphalt may result in higher exposure to PAHs and more adverse effects. Our aim is to assess occupational exposure to PAHs from conventional and CRM asphalt paving by measuring PAH metabolites in urine, and to investigate the effects on mitochondrial DNA copy number (mtDNAcn) and telomere length.

**Methods:**

We recruited 116 workers paving conventional asphalt, 51 workers paving CRM asphalt and 100 controls in Sweden, all males. A repeated-measures analysis included 31 workers paving both types of asphalt. Urine and blood samples were collected pre-working on Monday morning and post-working on Thursday afternoon after 4 days working. PAH metabolites: 1-hydroxypyrene (1-OH-PYR) and 2-hydroxyphenanthrene (2-OH-PH) were measured in urine by LC-MS/MS. Relative mtDNAcn and telomere length were measured by quantitative PCR.

**Results:**

Conventional and CRM asphalt workers showed higher 1-OH-PYR and 2-OH-PH than controls (*p* < 0.001 for all). Relative mtDNAcn were 0.21 units (*p* < 0.001) higher in conventional asphalt workers and 0.13 units (*p* = 0.010) higher in CRM asphalt workers compared to controls. Relative telomere length did not differ across occupational groups, but it was positively associated with increment of 2-OH-PH (β = 0.075, *p* = 0.037) in asphalt workers. The repeated-measures analysis showed no difference in either increment of 1-OH-PYP, or changes in effect biomarkers (mtDNAcn or telomere length) between paving with conventional and CRM asphalt. Increment of 2-OH-PH was smaller after paving with CRM asphalt.

**Conclusions:**

Road asphalt paving in open areas resulted in PAHs exposure, as shown by elevation of PAH metabolites in urine. Asphalt workers may experience oxidative stress, evidenced by alternation in mtDNAcn; however the effects could not be fully explained by exposure to PAHs from the asphalt mixture.

**Electronic supplementary material:**

The online version of this article (10.1186/s12940-018-0375-0) contains supplementary material, which is available to authorized users.

## Background

Hot asphalt emits a complex mixture of fumes, vapours and solid particulates containing hundreds of different compounds and carcinogens, such as polyaromatic hydrocarbons (PAHs), during paving application [1]. Many studies have been carried out to define the physical and chemical characteristics of asphalt fumes, as well as exposure levels and health effects in the work environment over the decades [2, 3], however, uncertainties still remained regarding exposure to asphalt fumes and related health effects due to the complexity of the asphalt fumes. Furthermore, studies have shown that skin uptake of components of the asphalt mixture is another important exposure pathway, especially for PAHs [4, 5]. Thus, asphalt workers are occupationally exposed to PAHs via both inhalation and dermal absorption [6]. It has been reported that exposure to asphalt mixture is related to increase in airway symptoms, decrease in lung function and increase in lung cancer incidence [3, 7, 8]. However, studies also found that it is difficult to disengage the role of asphalt exposure in itself from other potential exposures such as coal tar and smoking [9]. Recently, the International Agency for Research on Cancer (IARC) classified occupational exposures to conventional asphalt and its emissions during road paving as Group 2B (possibly carcinogenic to humans) [10]. Moreover, a technique of adding recycled crumb rubber to asphalt (crumb rubber modified asphalt, CRM asphalt) has been introduced to increase long-term performance and reduce traffic noise as compared to conventional asphalt (petroleum derived) [11]. Still, CRM asphalt paving could amplify toxic emissions due to higher processing temperatures (around 177 to 220 °C) and emissions from the crumb rubber additives [12]. Higher exposures to PAHs and particles during CRM asphalt work compared to conventional asphalt work have been described, as well as higher rates of acute irritation of eye, nose and throat in CRM asphalt workers than in conventional asphalt workers [13]. Experimental studies also showed a higher emission of particles from CRM asphalt than from conventional asphalt (Nilsson P, Bergendorf U, Tinnerberg H, Nordin E, Gustavsson M, Strandberg B, Albin M Gudmundsson. A Emissiond into the air from hitumen and rubber hitumen–implications for asphalt workers’ exposure, unpublished).

Oxidative stress is a widely accepted underlying mechanism between exposure to particles or chemicals and various adverse health outcomes. Lately, mitochondrial DNA (mtDNA) has become a biomarker of interest for oxidative stress. MtDNA lacks histones and has limited DNA repair capacity and it is therefore highly susceptible to oxidative DNA damage [14]. Reports have found that alteration of mtDNA content in peripheral blood can be related to toxic exposures, such as PAHs and benzene [15, 16], and mitochondria have been suggested to be a target for environmental pollutants [17]. Telomeres, as a tandem repeat sequence of TTAGGG located at the ends of the chromosomes that play a key role in chromosomal integrity, are also highly sensitive to oxidative stress [18, 19]. Changes in telomere length (TL) in peripheral blood have been related to different toxic exposures [20, 21]. Moreover, shorter TL seems to predict chromosomal instability [22] and the risk of several chronic diseases including cancer at several sites [23, 24], cardiovascular disease, diabetes and chronic obstructive pulmonary disease [25–27].

In this study, we assessed the current exposure to PAHs from both conventional and CRM asphalt by measuring PAH metabolites in urine to reflect a total internal dose of PAHs from inhalation and skin uptake; and investigated potential effects of exposure by measuring mtDNA copy number (mtDNAcn) and TL in peripheral blood. We also investigated if there was any difference in exposure and effects between conventional and CRM asphalt paving.

## Methods

### Study participants

The study population included 167 asphalt workers and 100 controls in Sweden. The asphalt workers were mainly employees of 4 large road construction and maintenance companies, and the workers were exposed to asphalt mixture when paving roads and pedestrian zones outdoors. There were 116 workers laying conventional asphalt and 51 laying CRM asphalt. For the conventional asphalt workers, the inclusion criterion was no exposure to CRM asphalt for at least 3 months. The controls worked with green area maintenance and were employees of the companies or municipal departments, performing manual outdoor work but with no known occupational exposure to asphalt mixture. All participants were male. The participants were investigated twice over 4 consecutive working days: pre-exposure on Monday morning (after a 72-h exposure-free period); and post-exposure on Thursday afternoon. The investigations took place from 2012 to 2015. The asphalt workers were investigated between April and October since this is the road paving season in Sweden; while around 50% of the controls were investigated between January and March due to availability. Among the asphalt workers laying CRM asphalt, 31 workers were also laying conventional asphalt occasionally. These workers were investigated twice (pre- and post-working) when laying CRM asphalt, and twice (pre- and post-working) when laying conventional asphalt at different period, therefore, a repeated-measures analysis was performed based on these 31 workers to compare conventional and CRM asphalt for biomarkers of exposure and toxicity.

All study participants gave informed written consent to take part in the study and the study was approved by the Regional Ethical Review Board in Lund, Sweden.

### Questionnaire investigation

One structured questionnaire was sent out to the participants one week before the field investigation. The questionnaire contained questions about working history (including potential particle exposure), medical history, smoking and smokeless tobacco “snus” (a moist snuff placed under the upper lip) history, medication, daily diet (fish, vegetable and fruit consumption), physical activity and recent respiratory symptoms. The questionnaire was handed in pre-working on Monday morning and was checked for omissions and inconsistencies during the examination by the occupational health nurse.

### Blood and urine sampling

First morning urine samples were collected pre-exposure on Monday morning, while spot urine samples were collected post-exposure on Thursday afternoon. All urine samples were transported to the laboratory at room temperature and stored at − 20 °C for further analysis.

Peripheral blood samples were obtained pre- and post-exposure onsite and transported to the laboratory on dry ice and stored at − 20 °C until extraction of DNA.

### Analysis of PAH metabolites in urine

We measured four PAH metabolites: 1-hydroxypyrene (1-OH-PYR), 2-hydroxyphenanthrene (2-OH-PH), 3-hydroxybenzo[a]pyrene (3-OH-BaP) and 3-hydroxy benz[a]anthracene (3-OH-BaA) from both pre- and post-working urine samples. 1-OH-PYR is a metabolite of pyrene and has been extensively used as a proxy for total exposure to PAH [28]. 2-OH-PH was chosen as the metabolite of phenanthrene, since asphalt fumes contains high concentration of phenanthrene [29]. It has been suggested that 2-OH-PH can be the most promising candidate of biomarkers of exposure to asphalt emissions [5]. 3-OH-BaP as metabolite of benzo[a]pyrene and 3-OH-BaA as metabolite of benz[a]anthracene were chosen since they can be found in asphalt fumes emission [29] and benzo(a)pyrene is classified as carcinogenic to human (IARC group 1) [30]. For the quantitative analysis, liquid chromatography coupled to tandem mass spectrometry was used (LC-MS/MS; QTRAP 5500, AB Sciex, Foster City, CA, USA). The urine samples were hydrolysed using glucuronidase and internal standards for all compounds were added. For analysis of 1-OH-PYR and 2-OH-PH, sample aliquots were injected into a single C18 column according to Svendsen et al. [31]. A two dimensional LC system was used for analysis of 3-OH-BaP and 3-OH-BaA. All samples were prepared in duplicates and the average concentrations were used. The analyses of 1-OH-PYR were part of a round robin inter-laboratory programme (University of Erlangen-Nuremberg, Germany) with results within the tolerance limits. Creatinine in urine was analysed using an enzymatic method and used for adjustment of urinary dilution (as μmol per mol creatinine) [32]. The limits of detection (LODs) were estimated from the blank samples and were 0.2 nmol/L for 1-OH-PYR and 2-OH-PH, and 0.05 nmol/L for 3-OH-BaP and 3-OH-BaA. Samples below LODs still have measured values, but with lower certainty. There were 79 (15%) samples below LOD for 1-OH-PYP and two (0.4%) samples below LOD for 2-OH-PH. The measured concentrations of the samples below LODs for 1-OH-PYP and 2-OH-PH were still used in statistical analysis. The concentrations of 3-OH-BaP and 3-OH-BaA were not evaluated since more than 95% of the samples were below the LODs. For more details on the method see Additional file 1.

### Analysis of relative quantitation of mitochondrial DNA copy number and telomere length in blood

Post-exposure peripheral blood samples were used to extract DNA for all participants. DNA was additionally extracted from pre-exposure samples from the 31 workers involved in the repeated-measures analysis. All DNA extractions were performed by using Qiagen DNA Blood Midi kit (Qiagen, Heidelberg, Germany).

As described previously, SYBR Green-based real-time quantitative PCR (7900HT, Applied Biosystems, Foster City, CA, USA) was used to determine relative mtDNAcn and TL [32]. Master mixes for mtDNA and hemoglobin beta (*HBB*) gene runs were prepared with KAPA SYBR FAST qPCR Kit Master Mix (2X) ABI Prism (Kapa Biosystems, Woburn, MA, USA) and corresponding primers (0.20 μM for each primer) [33, 34]. Master mixes for telomere runs were prepared with telomere primers (0.45 μM for each primer) [35], 1 × PCR Buffer (Thermo Fisher Scientific, Carlsbad, CA, USA), 1.75 mM MgCl_2_, 0.8 mM dNTPs, 0.3 mM SybrGreen (Thermo Fisher Scientific), 1 × Rox (Thermo Fisher Scientific), and 0.5 U *Taq* Platina (Thermo Fisher Scientific). The corresponding primers for mtDNA, telomere and *HBB* list in Additional file 2: Table S1. One reference DNA sample was diluted serially by twofold per dilution to produce 5 concentrations of 1–16 ng/μL for the standard curve. The standard curve, samples and one blank (sample with no template) were run in triplicates with 2.5 μl DNA (4 ng/μL) in each reaction. Each run was completed by melting curve analysis, and agarose gel electrophoresis of PCR products was performed for randomly picked samples to confirm the amplification specificity and absence of primer dimers.

R^2^ for each standard curve was > 0.99. Standard deviations of triplicates < 0.1 were accepted for the C_t_ values. SDS 2.4.1 software (Thermo Fisher Scientific) calculated the average relative quantity of triplicate measurements of mtDNAcn, TL and *HBB* for each sample based on the standard curve. Then, the average relative quantity of mtDNAcn was divided by the average quantity of *HBB* to calculate the mtDNA/*HBB* ratio (relative mtDNAcn). Likewise, the relative TL was the quotient of the average quantity of TL and *HBB*. Both relative mtDNAcn and TL are therefore arbitrary values. The CVs based on 4 runs were 9.1% for mtDNAcn and 7.5% for TL.

### Statistical analysis

Concentrations of PAH metabolites (1-OH-PYP and 2-OH-PH) were converted with natural logarithm to approach symmetric distribution and then included in model calculations. Relative mtDNAcn and TL were not converted since raw data showed close to symmetric distribution. Linear mixed model was used to compare urinary PAH metabolites across three occupational groups (conventional asphalt workers/CRM asphalt workers vs. controls) by adjusting for potential confounders (described below). General linear regression was used to analyze associations between exposure indexes [i.e. occupational groups as categorical variables, changes in urinary PAH metabolites (from pre- to post-working) as continuous variables] and post-working relative mtDNAcn or TL. Age (as continuous variable) was always included in the regression models. Other potential confounders [BMI (continuous variable), smoking and “snus” status (categorical variable), cigarette pack-year (continuous variable), investigation season (categorical variable), daily diet and physical activity (categorical variable)] were chosen based on published studies and general knowledge, and tested one by one in the models. Only the confounders which changed β-estimates of exposure indexes by more than 10% remained. Therefore, daily diet and physical activity were excluded.

In the repeated-measures analysis with 31 asphalt workers, absolute changes (Δ) from pre- to post-working of 1-OH-PYR, 2-OH-PH, relative mtDNAcn and TL were calculated for each subject for both conventional and CRM asphalt paving. Linear mixed models were adopted to analyze the differences in Δ values (i.e. ΔPAH metabolites, ΔmtDNAcn and ΔTL) between conventional and CRM asphalt paving; as well as the associations between ΔPAH metabolites and ΔmtDNAcn/ΔTL. Pre-working levels were included in the models for adjustment.

The residuals from each linear regression model were examined and all showed symmetric distribution. All statistical analyses were completed by using SPSS 23.0 (IBM SPSS Statistics for Windows, NY, USA).

## Results

**Fig. 1:**
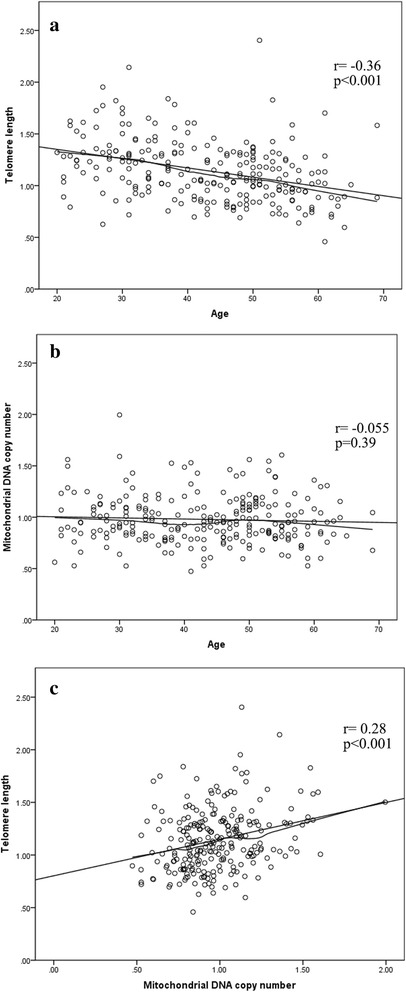
Correlations between telomere length, mitochondrial DNA copy number and age. Scatterplots with linear and loess fit lines showing that telomere length was inversely correlated with age (**a**), and positively correlated with mitochondrial DNA copy number (**c**). Mitochondrial DNA copy number was not correlated with age (**b**)

Basic characteristics of study participants are presented in Table 1. Age and BMI were similar across three occupational groups. The years of asphalt work did not differ between the conventional and the CRM asphalt groups. Smoking and snus status were slightly different: there were more current smokers in CRM asphalt workers (19%) compared to controls (12%); and more current snus users in asphalt workers (39%) compared to controls (21%); however smoking or snus were not correlated with either mtDNAcn or TL (data not show). The distributions of investigation season were similar between conventional asphalt and CRM asphalt workers (*p* = 0.56), but different to controls (*p* < 0.001) since 53% of controls were investigated during winter due to practical reason. Pre-working PAH metabolites were similar across three occupational groups, while post-working levels were different. Post-working relative mtDNAcn, but not relative TL were different across groups. Relative TL was inversely correlated with age (Fig. 1a), but relative mtDNAcn was not (Fig. 1b). TL and mtDNAcn were positively correlated (Fig. 1c). The distributions of daily diet and physical activity were similar across occupational groups, and neither of them was correlated with PAH metabolites or TL. Only daily diet was slightly correlated with mtDNAcn (Additional file 3: Table S2).

**Table 1:** Basic characteristics, PAH metabolites, mtDNAcn and TL for conventional asphalt workers, crumb rubber modified (CRM) asphalt workers and controls^a^

	Conventional asphalt workers (*n* = 116)	CRM asphalt workers (*n* = 51)	All asphalt workers (*n* = 167)	Controls (*n* = 100)	p^b^
Age	43 (24–59)	42 (22–61)	43 (23–59)	46 (24–62)	0.29
BMI (kg/m^2^)	28 (22–38)	28 (23–35)	28 (23–38)	27 (22–36)	0.89
Length of asphalt working (year)	12 (1–30)	10 (1–38)	12 (1–35)	–	–
Smoking					0.066
Never smoker	84 (74%)	31 (70%)	115 (73%)	65 (65%)	
Previous smoker	24 (21%)	5 (11%)	29 (18%)	23 (23%)	
Current smoker	6 (5%)	8 (19%)	14 (9%)	12 (12%)	
Cigarette pack-year if ever smoked	9 (2–30)	18 (1–95)	10 (1–38)	14 (1–54)	0.083
Snus					0.014
Never snus user	54 (47%)	25 (57%)	79 (50%)	66 (66%)	
Previous snus user	15 (13%)	2 (5%)	17 (11%)	13 (13%)	
Current snus user	45 (40%)	17 (38%)	62 (39%)	21 (21%)	
Investigation Season					< 0.001
Spring (Apr-Jun)	44 (38%)	17 (40%)	61 (39%)	26 (26%)	
Summer (Jul-Aug)	38 (33%)	10 (24%)	48 (30%)	18 (18%)	
Early autumn (Sep-Oct)	34 (29%)	15 (36%)	49 (31%)	3 (3%)	
Winter (Jan-Mar)	0 (0%)	0 (0%)	0 (0%)	53 (53%)	
Pre- working 1-OH-PYR (μmol/mol creatinine)	0.041 (0.018–0.11)	0.042 (0.011–0.17)	0.041 (0.017–0.13)	0.028 (0.0087–0.091)	0.12
Post- working 1-OH-PYR (μmol/mol creatinine)	0.068 (0.024–0.24)	0.11 (0.020–0.76)	0.076 (0.021–0.27)	0.028 (0.0092–0.091)	< 0.001
Pre- working 2-OH-PH (μmol/mol creatinine)	0.13 (0.063–0.55)	0.14 (0.061–0.51)	0.13 (0.063–0.52)	0.095 (0.040–0.65)	0.18
Post- working 2-OH-PH (μmol/mol creatinine)	0.20 (0.071–0.62)	0.24 (0.077–0.90)	0.21 (0.076–0.69)	0.082 (0.039–0.47)	< 0.001
Post-working relative mtDNAcn	0.98 (0.70–1.6)	0.97 (0.72–1.3)	0.98 (0.70–1.5)	0.87 (0.56–1.3)	< 0.001
Post-working relative TL	1.1 (0.76–1.6)	1.2 (0.73–1.8)	1.1 (0.73–1.7)	1.1 (0.72–1.7)	0.29

^a^Values are median (5–95 percentile) for continuous variables, or n (%) for categorical variables

^b^*P* values for age, BMI, cigarette pack-year, 1-OH-PYR, 2-OH-PH, relative mtDNA copy number and relative telomere length were derived from one-way ANOVA and *P* values for smoking, snus status and investigation season were derived from Fisher’s exact test to test the differences across three occupational groups

Ln(1-OH-PYP) and ln(2-OH-PH) (both pre- and post-working concentrations) showed a seasonal pattern in the conventional asphalt workers, indicating a trend of decreasing PAH exposure from spring to early autumn (*p* < 0.05 for all). No such pattern was shown in either CRM asphalt workers or controls. No seasonal pattern of ΔPAH metabolites during the working week was found, either (Additional file 4: Table S3). After adjusting for age, BMI, smoking and snus status and investigation season, both conventional and CRM asphalt workers showed higher ln(1-OH-PYR) and ln(2-OH-PH) than the controls (Table 2). The repeated-measures analysis showed that the increment of ln(2-OH-PH) was 0.50 μmol/mol creatinine smaller after paving with CRM asphalt compared to paving with conventional asphalt (95%CI: -0.85 – -0.15) after adjusting for pre-working concentration. No such difference between conventional and CRM asphalt paving was found for 1-OH-PYR increment (Table 3).

**Table 2:** Differences of urinary 1-OH-PYR and 2-OH-PH in three occupational groups^a^

PAH metabolites	Occupational groups	Adjusted mean (95%CI)	β (95% CI)	p
ln(1-OH-PYR)	Conventional asphalt workers	−2.6 (− 2.8, − 2.4)	0.76 (0.54, 0.97)	< 0.001
	CRM asphalt workers	−2.5 (− 2.8, − 2.3)	0.85 (0.58, 1.1)	< 0.001
	Control	−3.4 (− 3.6, − 3.2)	0	–
ln(2-OH-PH)	Conventional asphalt workers	−1.6 (− 1.8, − 1.5)	0.58 (0.36, 0.79)	< 0.001
	CRM asphalt workers	−1.6 (− 1.9, − 1.4)	0.58 (0.31, 0.85)	< 0.001
	Control	−2.2 (− 2.4, − 2.0)	0	–

^a^Adjusted mean, β estimates and *p* values were derived from linear mixed model after adjusting for age, BMI, smoking and snus status, cigarette pack-year and investigation season. The controls are the reference group

**Table 3:** Differences of changes (Δ) in urinary 1-OH-PYR and 2-OH-PH between conventional and CRM asphalt paving in the repeated-measures analysis (*N* = 31)^a^

ΔPAH metabolites	Type of asphalt paving	Adjusted mean (95%CI)	β (95% CI)	p
Δln(1-OH-PYR)	Conventional asphalt paving	0.86 (0.56, 1.2)	0	–
	CRM asphalt paving	0.86 (0.57, 1.2)	0.0068 (−0.37, 0.38)	0.97
Δln(2-OH-PH)	Conventional asphalt paving	0.88 (0.62, 1.1)	0	–
	CRM asphalt paving	0.38 (0.13, 0.62)	−0.50 (−0.85, − 0.15)	0.0073

^a^Adjusted mean, β estimates and *p* values were derived from linear mixed model after adjusting for pre-working PAH metabolites levels. Conventional asphalt paving are the reference group

Relative mtDNAcn, but not TL, was different between asphalt workers and controls (Table 4): mtDNAcn was 0.21 units (95%CI: 0.13–0.29) higher in conventional asphalt workers and 0.13 units (95%CI: 0.033–0.24) higher in CRM asphalt workers compared to controls. The analysis of dose-response associations between short-term exposure to PAH and relative mtDNAcn or TL among all asphalt workers showed that changes in PAH metabolites were positively associated with TL, but none of them was associated with mtDNAcn (Table 5). In the repeated-measures analysis, no difference was found in either change in relative mtDNAcn or change in TL from pre- to post-working among the same workers paving with conventional and CRM asphalt (Additional file 5: Table S4). However, a negative association was found between change in 1-OH-PYP and change in mtDNAcn during the working week among 31 workers in the repeated-measures analysis (Table 5), indicating that a short-term higher exposure to pyrene from asphalt mixture was associated with less increment of mtDNAcn (β = − 0.078, 95%CI: -0.14 – -0.016).

**Table 4:** Differences of post-working relative mtDNAcn and TL in three occupational groups

	Post-working mtDNAcn				Post-working TL			
	Partly adjusted^a^		Fully adjusted^b^		Partly adjusted^a^		Fully adjusted^b^	
Occupational groups	β (95% CI)	p	β (95% CI)	p	β (95% CI)	p	β (95% CI)	p
Conventional asphalt	0.14 (0.076, 0.20)	< 0.001	0.21 (0.13, 0.29)	< 0.001	−0.029 (− 0.10, 0.046)	0.45	− 0.017 (− 0.12, 0.085)	0.74
CRM asphalt	0.084 (0.0018, 0.17)	0.045	0.13 (0.033, 0.24)	0.010	0.045 (−0.054, 0.14)	0.37	0.064 (−0.065, 0.19)	0.33
Controls	0	–	0	–	0	–	0	–
All asphalt workers	0.12 (0.065, 0.18)	< 0.001	0.20 (0.12, 0.28)	< 0.001	−0.0084 (− 0.079, 0.062)	0.82	0.0011 (− 0.098, 0.10)	0.98
Controls	0	–	0	–	0	–	0	–

^a^Partly adjusted linear regression model: only adjusted for age

^b^Fully adjusted linear regression model: adjusted for age, BMI, smoking and snus status, cigarette pack-year and investigation season

**Table 5:** Associations between changes (Δ) in PAH metabolites and post-working relative mtDNAcn / TL among asphalt workers; and ΔmtDNAcn / ΔTL in the repeated-measures analysis

Study population	ΔPAH metabolites	Post-working relative mtDNAcn		Post-working relative TL	
		β (95%CI)	p^a^	β (95%CI)	p^a^
All asphalt workers	Δln(1-OH-PYR)	0.035 (−0.028, 0.098)	0.28	0.067 (−0.0038, 0.14)	0.064
(*N* = 138)	Δln(2-OH-PH)	0.048 (−0.015, 0.11)	0.14	0.075 (0.0046, 0.15)	0.037
		Δ mtDNAcn		Δ TL	
		β (95%CI)	p^b^	β (95%CI)	p^b^
Repeated-measures analysis	Δln(1-OH-PYR)	−0.078 (− 0.14, − 0.016)	0.015	−0.064 (− 0.16, 0.028)	0.17
(N = 31)	Δln(2-OH-PH)	−0.057 (− 0.12, 0.0073)	0.080	− 0.048 (− 0.15, 0.049)	0.32

^a^*P* values were derived from general linear model with age, BMI, smoking and snus states, cigarette pack-year, investigation season, and occupational groups as adjustments

^b^*P* values were derived from linear mixed model with occupational groups and pre-working mtDNAcn or TL as adjustments

## Discussion

Road paving asphalt workers, both paving with conventional and CRM asphalt are exposed to PAHs, evidenced by higher 1-OH-PYR and 2-OH-PH in the workers. However, the exposure levels were not as high as in other studies of asphalt workers [36, 37]. Asphalt workers seem to experience moderate oxidative stress from this occupation, as indicated by higher relative mtDNAcn than controls; however, such alternation could not be fully explained by PAHs from asphalt mixture.

In this study, we measured urinary PAH metabolites to reflect the total internal dose of PAHs from inhalation and dermal exposure, and our observations of clear increases in the PAH metabolites among the exposed workers but not among the controls are in accordance with other studies of asphalt paving workers [28, 38]. However, it should be noted that 1-OH-PYR concentrations in the present study were much lower than reported in the other studies on asphalt workers performed in different countries in Europe [36, 37]. The relatively low PAHs exposure can also be seen in the air measurements in our study (median concentrations for total airborne PAHs were around 2.8 and 2.6 μg/m^3^ for conventional and CRM asphalt, respectively) (Xu Y, Kåredal M, Nielsen J, Adlercreutz M, Bergendorf U, Strandberg B, Antonsson AB, Tinnerberg H, Albin M: Exposure, respiratory symptoms, lung function and inflammation response of road paving asphalt workers. Submitted), which were lower than the current threshold limit value (TLV: 200 μg/m^3^) set by the American Conference of Governmental Industrial Hygienists (ACGIH) [39]. A smaller increment of 2-OH-PH after CRM asphalt paving was noted among 31 workers in the repeated-measures analysis, indicating that the workers were exposed to less phenanthrene when paving with CRM asphalt than with conventional asphalt. Such finding is different from U.S. studies reporting higher PAH emissions from CRM paving [13, 40] and our laboratory measurements of our project (Nilsson P, Bergendorf U, Tinnerberg H, Nordin E, Gustavsson M, Strandberg B, Albin M, Gudmundsson A. Emissions into the air from bitumen and rubber bitumen–implications for asphalt workers’ exposure. Unpublished). The contradictory findings may due to the modest sample size in our study. Besides, paving temperature could be a more important determinate factor of PAH emissions. It has been shown that paving at higher temperatures is associated with higher exposure levels [3, 41].

Given the fact that mtDNA and telomeres are highly sensitive to damage by oxidative stress, together with the evidence that both mtDNAcn and TL may be influenced by short-term particle exposure [42, 43], we hypothesized that these two biomarkers are dynamic and can be affected by short-term exposure to PAHs. Higher relative mtDNAcn in asphalt workers may indicate moderate oxidative stress in this occupation. The asphalt workers may hypothetically experience a compensatory response: mtDNA synthesis is stimulated to produce more energy for damaged mitochondria disposal and cell survival under low to moderate oxidative stress [44]. Earlier studies on exposure to PAHs and response of mtDNA have shown conflicting results. Pavanello S et al. found a positive association between urinary 1-OH-PYP and mtDNAcn among cokeoven workers [15]. Kim HY et al. reported increased mtDNAcn in human leukaemia-derived cell lines and bone marrow-derived mesenchymal stem cells after PAHs treatment at a concentration of 100 μM [45]. However, Pieters N et al. showed decreased mtDNAcn in association with indoor exposure to PAHs during wintertime [46]. One reason for these conflicting results could be that different exposure levels and exposure length can result in different responses in the body. The dose-response of short-term PAH exposure on mtDNAcn was uncertain. We did not find associations between changes in PAH metabolites and post-working mtDNAcn among all asphalt workers. However, we found a higher increment of 1-OH-PYP in association with less increment in mtDNAcn during four days among 31 workers in the repeated-measures analysis. One explanation could be that other compounds than PAHs in the asphalt mixture, e.g. nitrosamines, or unmeasured co-exposures, e.g. traffic pollutions, were truly responsible for the increased mtDNAcn, while the short-term effect (repeated-measures analysis) of PAH could, as an acute response, be associated with decreased mtDNAcn.

The dose-response effect of short-term PAHs exposure on relative TL showed positive associations between changes in 1-OH-PYR and 2-OH-PH and post-working TL, but no association with change in TL during four days in the repeated-measures analysis. One may infer that the short-term PAHs exposure has no effect on TL within four days, or our study was not powerful enough to detect the difference. Yet we could not rule out the possibility that it may affect TL as a long-term effect. Studies of coke-oven workers in Chinese and European populations reported short TL in related to PAHs exposure [20, 47]. The exposure levels in those studies were much higher (median urinary 1-OH-PYR: 12.2 μmol/mol creatinine in Chinese study and 3.09 in European study) than in our study (0.08 for all asphalt workers), indicating the possible TL shortening effect of PAHs exposure may only happen at higher exposure level. Instead, short-term exposure to low level of PAHs in asphalt workers may induce acute inflammatory response [48], which can cause influx of “younger” inflammatory cells with longer TL to the blood or activation of telomerase to elongate TL [49], which may take place during weeks.

There were some limitations with our study. The number of workers involved in paving with CRM asphalt was small and may have limited our possibilities to discern differences in exposure and effects as compared to conventional asphalt paving. Asphalt paving has complex exposure scenarios including other established carcinogens (e.g. nitrosamines) and small combustion particles. Although we performed air sampling of about 50 paving workers to monitor air-borne nitrosamines and respirable dust (Xu Y, Kåredal M, Nielsen J, Adlercreutz M, Bergendorf U, Strandberg B, Antonsson AB, Tinnerberg H, Albin M: Exposure, respiratory symptoms, lung function and inflammation response of road paving asphalt workers. Submitted), we could not estimate exposure levels for these co-exposures for all workers due to outdoor work complexity and large variations of asphalt mix and paving temperature, and therefore we were not able to address any associations between exposures other than PAHs and mtDNAcn or TL. The investigation season was different from asphalt workers and controls due to practical reason. As sensitivity analysis, we excluded study participants investigated in winter to improve the consistency of investigation season. The effect estimates and *p* values were similar as the original analysis (Additional file 6: Table S5), which lends credibility to our results. On the other hand, the present study had several strengths. We prolonged the exposure period to more than a single day’s working-shift. The purpose was to include the exposure and effect from the working week rather than only a single working day, and this could supposedly reduce the day-to-day variation.

## Conclusions

Our results are in agreement with previous studies showing that asphalt workers are still exposed to PAHs. However, the exposure levels were lower than in previous studies. Still, it seems that exposure to asphalt mixture can induce oxidative stress, as evidenced by higher relative mtDNAcn in the asphalt workers; however the associations found for mtDNAcn cannot be fully explained by exposure to PAHs from asphalt mixture. Other unavailable co-exposures during asphalt paving may have effects apart from that of PAHs, and would weaken our possibilities to observe associations. We did not find any evidence of higher PAHs exposure or more effects on mtDNAcn and TL when working with CRM asphalt compared to conventional asphalt. Paving temperature could be a more important factor than crumb rubber additives with respect of exposure and related effects.

## Additional files

Additional file 1: Measurement of 1-OH-PYR, 2-OH-PH, 3-OH-BaA and 3-OH-BaP in urine (DOCX 21 kb)

Additional file 2: **Table S1.** Real Time PCR primers for mtDNA, telomere and hemoglobin beta (*HBB*) (DOCX 17 kb)

Additional file 3: **Table S2.** Distribution of daily diet and physical activity across occupational groups and their correlations with urinary PAH metabolites, mtDNAcn and TL (DOCX 21 kb)

Additional file 4: **Table S3.** Pre-, post-working and changes (Δ) in PAH metabolites in three occupational groups investigated in different investigation season. (DOCX 24 kb)

Additional file 5: **Table S4.** Differences of changes (Δ) in relative TL and mtDNAcn between conventional and CRM asphalt paving in the repeated-measures analysis (*N* = 31) (DOCX 21 kb)

Additional file 6: **Table S5.** Sensitivity analysis of PAH exposures and biomarkers in three groups, excluding participants from winter. (DOCX 22 kb)

## Abbreviations

1-OH-PYR: 1-hydroxypyrene
2-OH-PH: 2-hydroxyphenanthrene
3-OH-BaA: 3-hydroxybenz[a]anthracene
3-OH-BaP: 3-hydroxy benso[a]pyrene
CRM: Crumb rubber modified
*HBB*: Hemoglobin beta
LODs: Limits of detection
mtDNA: mitochondrial DNA
mtDNAcn: mitochondrial DNA copy number
PAHs: Polyaromatic hydrocarbons
PCR: Polymerase chain reaction
TL: Telomere length
