# Supplementary material for: Occupational exposure to asphalt mixture during road paving is related to increased mitochondria DNA copy number: a cross-sectional study
Source: Environ Health. 2018 Mar 27;17:29. doi: 10.1186/s12940-018-0375-0 (PMC5870390; doi:10.1186/s12940-018-0375-0)
Supplement: Supplementary file 1 — Measurement of 1-OH-PYR, 2-OH-PH, 3-OH-BaA and 3-OH-BaP in urine (DOCX 21 kb) [file 12940_2018_375_MOESM1_ESM.docx]

**Measurement of 1-OH-PYR, 2-OH-PH, 3-OH-BaA and 3-OH-BaP in urine**

Chemicals

1-hydroxypyrene (1-OH-PYR), 2-hydroxyphenanthrene (2-OH-PH), 3-hydroxybenzo[a]anthracene (3-OH-BaA), 3-hydroxybenzo[a]pyrene (3-OH-BaP) and the internal standards  ^2^H_9_-1-OH-PYR, ^2^H_9_-2-OH-PH , ^2^H_11_-3-OH-BaA and ^2^H_11_-3-OH-BaP were obtained from Toronto Research Chemicals, Inc. (North York, Canada). Ammonium acetate and methanol were from Merck (Darmstadt, Germany). Water was from a Milli-Q Integral 5 system (Millipore, Billerica, MA, USA). β-Glucuronidase (*Escherichia coli* K12) was obtained from Roche Diagnostics (Mannheim, Germany).

Urine samples for calibration and for quality control (QC) were obtained from healthy volunteers at our laboratory and exposed subjects. The two QC samples for 1-OH-PYR had concentrations of 6 and 26 nmol/L, respectively, and the two QC samples for 2-OH-PH had concentrations of 20 and 95 nmol/L, respectively.

Instrumentation

For the quantitative analysis a triple quadrupole linear ion trap mass spectrometer equipped with an electrospray ion source (QTRAP 5500; AB Sciex, Foster City, CA, USA) coupled to a liquid chromatograph with four pumps (LC-MS/MS; Shimadzu Corporation, Kyoto, Japan). Air was used as nebulizer and auxiliary gas, pure nitrogen as curtain and collision gas. The MS analyses were carried out by selected reaction monitoring in the negative mode (Table S1).

Sample preparation

For analysis of PAH metabolites, 200 µL duplicate urine samples were placed in 1 mL micro inserts in 96-well plates, and 10 µL glucuronidase and 100 µL 1 mol/L ammonium acetate buffer, pH 6.5, were added. The samples were kept at 37 °C for 30 min. After incubation, 40 ul vitamin C solution of 0.18 mg/ml and an IS solution containing ^2^H_9_-1-OH-PYR, ^2^H_9_-2-OH-PH, ^2^H_11_-3-OH-BaA and ^2^H_11_-3-OH-BaP were added. The samples were stored at -20 °C until analysis. The samples were shaken and centrifuged at 3000 rpm for 10 min just prior to analysis.

Instrumental analysis

For analysis of 1-OH-PYR and 2-OH-PH, sample aliquots of 5 µL were injected onto a C18 column (Genesis Lightn, 2.1 mm i.d. x 100 mm, Genesis, Grace Vydac, Hesperia, CA, USA) kept at 60 °C. A mobile phase gradient, flow rate 0.3 mL/min, consisting of water (A) and methanol (B) was kept at 5% B for 1 min after injection, raised to 95% B in 6.7 min B, kept there for 2.3 min; for the next injection, the column was conditioned at 5% B for 3 min. A diverter valve was used to introduce the column effluent into the mass spectrometer between 5.8 and 6.9 min. The ion source temperature was 700 °C, for other parameters see Table S1.

In the analysis of benso[a]pyrene and benso[a]antracene, the sum of several hydroxylated metabolites were quantified as 3-OH-BaP and 3-OH-BaA equivalents, respectively. For analysis of 3-OH-BaA and 3-OH-BaP a two dimensional separation was carried out, using two analytical columns; Column A: Genesis (C8, 4.6 × 20 mm, 4 μm) and column B: Genesis Lightn (C18, 4.6 × 100 mm, 4 μm,) and four LC pumps. The columns and LC pumps were connected through a diverter valve. The two mobile phases used consisted of water (A) and methanol (B). An aliquot of 20 μL of the sample was injected on column A and the separation was carried out by gradient elution, beginning with 55% mobile phase B for 1.55 minutes and a gradient to 70% B for 1.5 minutes. After 2.85 min, the diverter valve switched over and the effluent was diverted onto column B during 1.3 min. The second set of pumps continued the gradient from 70% B to 95% B for 2 minutes on column B. A diverter valve on the MS diverted the column B effluent to the MS between 4.5–6.3 min. Column A was reconditioned with 95% mobile phase B for 1 min, followed by equilibration with 55% mobile phase A for 2.5 min and column B was reconditioned with 95% mobile phase B for 1 min in the end of the analytical run and then equilibrated with 70% mobile phase B during the beginning of the next analytical run. The columns were maintained at 60^◦^C and the flow rate was 0.6 mL/min. The ion source temperature was 700 °C, other parameters see Supplementary table 1.

Data were acquired and processed using the supplied software (Analyst 1.6.1, Multiquant 2.1, AB Sciex). Concentrations were determined by peak area ratios of the analytes versus the ISs. Within each analytical batch, 80 urine samples, a calibration curve, 2 QC samples for 1-OH-PYR and 2-OH-PH and 2 chemical blanks were included. All samples were prepared in duplicates and analyzed by single injections. The average concentration of the duplicate samples was used.

Validation

The limits of detection (LODs) were estimated from the blank samples and were 0.2 nmol/L for 1-OH-PYR and 2-OH-PH, and 0.05 nmol/L for 3-OH-BaP and 3-OH-BaA. Analytical reproducibility, expressed as coefficient of variation (CV) in n = 400 duplicate urine samples, was 14% for 1-OH-PYR at 2 nmol/L, and 9% for 2-OH-PH at 4 nmol/L.

The between-run precision determined for 1-OH-PYR was determined from 2 QC samples and was 8% at the levels 6 and 26 nmol/L**.** The QC samples were analysed 32 times during 11 months. The between-run precision for 2-OH-PH was determined from 2 QC samples and was 9% at the levels 20 and 95 nmol/L. These QC samples were analysed 25 times during 8 months. The analyses of 1-OH-PYR were part of a round robin inter-laboratory programme (University of Erlangen-Nuremberg, Germany) with results within the tolerance limits.

Table S1: SRM transitions for 1-OH-PYR, 2-OH-PH, 3-OH-BaA and 3-OH-BaP in LC-MS/MS analysis including declustering potentials (DP) and collision energies (CE).

| **Analyte** | **Transitions**  **[m/z]** |  | **DP**  **(V)** | **CE**  **(eV)** |
| --- | --- | --- | --- | --- |
| 1-OH-PYR | 217 - 189 | *Quantifier ion* | -140 | -47 |
|  |  |  |  |  |
| ^2^H_9_-1-OH-PYR | 226 - 198 | *IS* | -140 | -47 |
|  |  |  |  |  |
| 2-OH-PH | 193 - 165 | *Quantifier ion* | -140 | -46 |
|  |  |  |  |  |
| ^2^H_9_-2-OH-PH | 203 - 174 | *IS* | -140 | -46 |
|  |  |  |  |  |
| 3-OH-BaA | 243 - 215 | *Quantifier ion* | -140 | -47 |
|  |  |  |  |  |
| ^2^H_11_-3-OH-BaA | 254 - 226 | *IS* | -140 | -47 |
|  |  |  |  |  |
| 3-OH-BaP | 267 - 239 | *Quantifier ion* | -140 | -54 |
|  |  |  |  |  |
| ^2^H_11_-3-OH-BaP | 278 - 250 | *IS* | -140 | -54 |
