# Supplementary material for: Occupational exposure to asphalt mixture during road paving is related to increased mitochondria DNA copy number: a cross-sectional study
Source: Environ Health. 2018 Mar 27;17:29. doi: 10.1186/s12940-018-0375-0 (PMC5870390; doi:10.1186/s12940-018-0375-0)
Supplement: Supplementary file 2 — Table S1. Real Time PCR primers for mtDNA, telomere and hemoglobin beta (HBB) (DOCX 17 kb) [file 12940_2018_375_MOESM2_ESM.docx]

Table S1: Real Time PCR primers for mtDNA, telomere and hemoglobin beta (*HBB*)

| Genome | Forward Primer Seq 5′-3′ | Reverse Primer Seq 5′-3′ | Amplicon (bps) | References |
| --- | --- | --- | --- | --- |
| MtDNA | CAC CCA AGA ACA GGG TTT GT | TGG CCA TGG GTA TGT TGT TA | 107 | [33, 34] |
| Telomere | CGG TTT GTT TGG GTT TGG GTT TGG GTT TGG GTT TGG GTT | GGC TTG CCT TAC CCT TAC CCT TAC CCT TAC CCT TAC CT | 76 | [35] |
| *HBB* | TGT GCT GGC CCA TCA CTT TG | ACC AGC CAC CAC TTT CTG ATA GG | 73 | [33] |
