# Supplementary material for: Occupational exposure to asphalt mixture during road paving is related to increased mitochondria DNA copy number: a cross-sectional study
Source: Environ Health. 2018 Mar 27;17:29. doi: 10.1186/s12940-018-0375-0 (PMC5870390; doi:10.1186/s12940-018-0375-0)
Supplement: Supplementary file 3 — Table S2. Distribution of daily diet and physical activity across occupational groups and their correlations with urinary PAH metabolites, mtDNAcn and TL (DOCX 21 kb) [file 12940_2018_375_MOESM3_ESM.docx]

Table S2. Distribution of daily diet and physical activity across occupational groups and their correlations with urinary PAH metabolites or mtDNA copy number, TL

| Diet | | three groups | | | | | | | | | P for  distribution | P for  correlation  with  1-OH-PYP | P for  correlation  with  2-OH-PH | P for  correlation  with mtDNA copy number | P for  correlation  with TL |
| --- | --- | --- | --- | --- | --- | --- | --- | --- | --- | --- | --- | --- | --- | --- | --- |
|  |  | Conventional asphalt workers | | | CRM asphalt workers | | | | Controls | |  |  |  |  |  |
|  |  | Count | | % | Count | | % | | Count | % |  |  |  |  |  |
| vegetable consumption | every day | 53 | | 46.5% | 18 | | 40.9% | | 59 | 59.0% | 0.13 | 0.66 | 0.66 | 0.036 | 0.12 |
|  | several times/week | 50 | | 43.9% | 19 | | 43.2% | | 36 | 36.0% |  |  |  |  |  |
|  | several times/month | 9 | | 7.9% | 5 | | 11.4% | | 5 | 5.0% |  |  |  |  |  |
|  | never | 2 | | 1.8% | 2 | | 4.5% | | 0 | 0.0% |  |  |  |  |  |
| fruit consumption | every day | 45 | | 39.5% | 12 | | 27.3% | | 60 | 60.0% | 0.069 | 0.20 | 0.44 | 0.016 | 0.68 |
|  | several times/week | 56 | | 49.1% | 22 | | 50.0% | | 34 | 34.0% |  |  |  |  |  |
|  | several times/month | 10 | | 8.8% | 9 | | 20.5% | | 6 | 6.0% |  |  |  |  |  |
|  | never | 3 | | 2.6% | 1 | | 2.3% | | 0 | 0.0% |  |  |  |  |  |
| fish consumption | every day | 2 | | 1.8% | 2 | | 4.5% | | 0 | 0.0% | 0.44 | 0.41 | 0.21 | 0.076 | 0.97 |
|  | several times/week | 59 | | 51.8% | 23 | | 52.3% | | 59 | 59.0% |  |  |  |  |  |
|  | several times/month | 38 | | 33.3% | 13 | | 29.5% | | 26 | 26.0% |  |  |  |  |  |
|  | never | 15 | | 13.2% | 6 | | 13.6% | | 15 | 15.0% |  |  |  |  |  |
| Physical activity | |  | | | | | | | | | | | | | |
| Activity or training | No activity | 15 | 13.2% | | | 3 | | 6.8% | 16 | 16.0% | 0.068 | 0.31 | 0.12 | 0.14 | 0.98 |
|  | Little activity | 57 | 50.0% | | | 24 | | 54.5% | 37 | 37.0% |  |  |  |  |  |
|  | Normal activity | 29 | 25.4% | | | 11 | | 25.0% | 19 | 19.0% |  |  |  |  |  |
|  | Intensive activity | 13 | 11.4% | | | 6 | | 13.6% | 28 | 28.0% |  |  |  |  |  |

P for distribution derived from Fisher’s exact test.

P for correlation derived from Spearman’s correlation test.
