# Supplementary material for: Occupational exposure to asphalt mixture during road paving is related to increased mitochondria DNA copy number: a cross-sectional study
Source: Environ Health. 2018 Mar 27;17:29. doi: 10.1186/s12940-018-0375-0 (PMC5870390; doi:10.1186/s12940-018-0375-0)
Supplement: Supplementary file 4 — Table S3. Pre-, post-working and changes (Δ) in PAH metabolites in three occupational groups investigated in different investigation season. (DOCX 24 kb) [file 12940_2018_375_MOESM4_ESM.docx]

Table S3: Pre-, post-working and change (Δ) in PAH metabolites in three occupational groups investigated in different season.

|  |  | Conventional asphalt workers | | | CRM asphalt workers | | | Controls | | |
| --- | --- | --- | --- | --- | --- | --- | --- | --- | --- | --- |
|  |  | N | Adjusted Mean  (95% CI) ^a^ | p ^b^ | N | Adjusted Mean  (95% CI) ^a^ | p ^b^ | N | Adjusted Mean  (95% CI) ^a^ | p ^b^ |
| Pre-working  ln(1-OH-PYP) | Winter | 0 | -- | 0.0030 | 0 | -- | 0.12 | 51 | 0.049 (0.031, 0.068) | 0.62 |
|  | Spring | 42 | 0.080 (0.062, 0.098) |  | 17 | 0.081 (0.044, 0.12) |  | 24 | 0.035 (0.011, 0.060) |  |
|  | Summer | 36 | 0.055 (0.036, 0.073) |  | 6 | 0.082 (0.039, 0.13) |  | 18 | 0.038 (0.011, 0.065) |  |
|  | Autumn | 29 | 0.053 (0.032, 0.074) |  | 15 | 0.046 (0.0072, 0.085) |  | 3 | 0.033 (-0.032, 0.090) |  |
| Post-working  ln(1-OH-PYP) | Winter | 0 | -- | 0.0032 | 0 | -- | 0.19 | 52 | 0.049 (0.031, 0.067) | 0.93 |
|  | Spring | 42 | 0.16 (0.13, 0.18) |  | 17 | 0.30 (0.16, 0.44) |  | 24 | 0.053 (0.029, 0.077) |  |
|  | Summer | 36 | 0.14 (0.12, 0.17) |  | 6 | 0.19 (0.028, 0.36) |  | 18 | 0.045 (0.019, 0.071) |  |
|  | Autumn | 29 | 0.11 (0.085, 0.14) |  | 15 | 0.18 (0.034, 0.33) |  | 3 | 0.041 (-0.014, 0.096) |  |
| Pre-working  ln(2-OH-PH) | Winter | 0 | -- | 0.035 | 0 | -- | 0.58 | 51 | 0.16 (0.081, 0.24) | 0.94 |
|  | Spring | 42 | 0.23 (0.15, 0.31) |  | 17 | 0.23 (0.14, 0.33) |  | 24 | 0.17 (0.064, 0.78) |  |
|  | Summer | 36 | 0.15 (0.070, 0.23) |  | 6 | 0.19 (0.078, 0.30) |  | 18 | 0.14 (0.021, 0.25) |  |
|  | Autumn | 29 | 0.14 (0.057, 0.23) |  | 15 | 0.19 (0.089, 0.29) |  | 3 | 0.12 (-0.12, 0.36) |  |
| Post-working  ln(2-OH-PH) | Winter | 0 | -- | 0.0018 | 0 | -- | 0.066 | 52 | 0.16 (0.11, 0.20) | 0.65 |
|  | Spring | 42 | 0.37 (0.29, 0.45) |  | 17 | 0.48 (0.29, 0.67) |  | 24 | 0.19 (0.13, 0.25) |  |
|  | Summer | 36 | 0.30 (0.21, 0.38) |  | 6 | 0.24 (0.016, 0.47) |  | 18 | 0.16 (0.092, 0.23) |  |
|  | Autumn | 29 | 0.22 (0.13, 0.31) |  | 15 | 0.28 (0.080, 0.49= |  | 3 | 0.13 (-0.0091, 0.27) |  |
| Δln(1-OH-PYP) | Winter | 0 | -- | 0.12 | 0 | -- | 0.32 | 51 | 0 (-0.014, 0.015) | 0.35 |
|  | Spring | 40 | 0.078 (0.052, 0.10) |  | 17 | 0.22 (0.087, 0.35) |  | 24 | 0.017 (-0.0020, 0.037) |  |
|  | Summer | 36 | 0.088 (0.061, 0.11) |  | 6 | 0.11 (-0.047, 0.27) |  | 18 | 0.0062 (-0.015, 0.028) |  |
|  | Autumn | 29 | 0.061 (0.031, 0.090) |  | 15 | 0.14 (-0.005, 0.28) |  | 3 | 0.0071 (-0.037, 0.052) |  |
| Δln(2-OH-PH) | Winter | 0 | -- | 0.23 | 0 | -- | 0.095 | 51 | -0.0025 (-0.058, 0.053) | 0.90 |
|  | Spring | 40 | 0.14 (0.052,0.22) |  | 17 | 0.25 (0.086, 0.41) |  | 24 | 0.021 (-0.054, 0.095) |  |
|  | Summer | 36 | 0.15 (0.065, 0.24) |  | 6 | 0.054 (-0.14, 0.25) |  | 18 | 0.022 (-0.060, 0.10) |  |
|  | Autumn | 29 | 0.080 (-0.016, 0.18) |  | 15 | 0.095 (-0.079, 0.27) |  | 3 | 0.012 (-0.16, 0.18) |  |

a Adjusted mean were derived from general linear regression after adjusting for age, BMI, smoking and snus status, cigarette pack-year

b P values were between season effects, derived from adjusted general linear regression
