# Supplementary material for: Occupational exposure to asphalt mixture during road paving is related to increased mitochondria DNA copy number: a cross-sectional study
Source: Environ Health. 2018 Mar 27;17:29. doi: 10.1186/s12940-018-0375-0 (PMC5870390; doi:10.1186/s12940-018-0375-0)
Supplement: Supplementary file 5 — Table S4. Differences of changes (Δ) in relative TL and mtDNAcn between conventional and CRM asphalt paving in the repeated-measures analysis (N = 31) (DOCX 21 kb) [file 12940_2018_375_MOESM5_ESM.docx]

Table S4: Differences of changes (Δ) in relative TL and mtDNAcn between conventional and CRM asphalt paving in the repeated-measures analysis (N=31)

| Biomarkers | Type of asphalt paving | Adjusted mean (95%CI) | β (95% CI) | p ^b^ |
| --- | --- | --- | --- | --- |
| ΔTL ^a^ | Conventional asphalt paving | 0.34 (0.24, 0.44) | 0 | -- |
|  | CRM asphalt paving | 0.31 (0.20, 0.41) | -0.032 (-0.15, 0.084) | 0.58 |
| ΔmtDNAcn ^a^ | Conventional asphalt paving | 0.24 (0.16, 0.32) | 0 | -- |
|  | CRM asphalt paving | 0.21 (0.12, 0.29) | -0.032 (-0.11, 0.044) | 0.40 |

^a^ ΔTL = TL (post-working) – TL (pre-working); ΔmtDNAcn = mtDNAcn (post-working) – mtDNAcn (pre-working)

^b^ P values were derived from linear mixed model with pre-working values as adjustments.
