# Supplementary material for: Occupational exposure to asphalt mixture during road paving is related to increased mitochondria DNA copy number: a cross-sectional study
Source: Environ Health. 2018 Mar 27;17:29. doi: 10.1186/s12940-018-0375-0 (PMC5870390; doi:10.1186/s12940-018-0375-0)
Supplement: Supplementary file 6 — Table S5. Sensitivity analysis of PAH exposures and biomarkers in three groups, excluding participants from winter. (DOCX 22 kb) [file 12940_2018_375_MOESM6_ESM.docx]

Table S5. Sensitivity analysis of PAH exposures ^a^ and biomarkers ^b^ in three groups, excluding participants from winter.

| PAH metabolites | Occupational groups | Including all (original analysis) | | | Excluding subjects investigated in winter | | |
| --- | --- | --- | --- | --- | --- | --- | --- |
|  |  | N | β (95% CI) | p | N | β (95% CI) | p |
| Ln(1-OH-PYR) | Conventional asphalt workers | 116 | 0.76  (0.54, 0.97) | <0.001 | 116 | 0.67  (0.46, 0.88) | <0.001 |
|  | CRM asphalt workers | 42 | 0.85  (0.58, 1.1) | <0.001 | 42 | 0.72  (0.47, 0.98) | <0.001 |
|  | Control | 100 | 0 | -- | 45 | 0 | -- |
| Ln(2-OH-PH) | Conventional asphalt workers | 116 | 0.58  (0.36, 0.79) | <0.001 | 116 | 0.51  (0.30, 0.72) | <0.001 |
|  | CRM asphalt workers | 42 | 0.58  (0.31, 0.85) | <0.001 | 42 | 0.52  (0.26, 0.78) | <0.001 |
|  | Control | 100 | 0 | -- | 45 | 0 | -- |
| Biomarkers |  |  |  |  |  |  |  |
| mtDNAcn | Conventional asphalt workers | 116 | 0.21  (0.13, 0.29) | <0.001 | 116 | 0.21  (0.13, 0.30) | <0.001 |
|  | CRM asphalt workers | 42 | 0.13  (0.033, 0.24) | 0.010 | 42 | 0.11  (0.006, 0.21) | 0.037 |
|  | Control | 100 | 0 | -- | 45 | 0 | -- |
| TL | Conventional asphalt workers | 116 | -0.017  (-0.12, 0.085) | 0.74 | 116 | -0.010  (-0.105, 0.084) | 0.83 |
|  | CRM asphalt workers | 42 | 0.064  (-0.065, 0.19) | 0.33 | 42 | 0.070  (-0.045, 0.19) | 0.23 |
|  | Control | 100 | 0 | -- | 45 | 0 | -- |

^a^ Linear mixed model was used with adjusting for age, BMI, smoking and snus status, cigarette pack-year and investigation season. The controls are the reference group.

b General linear regression was used with adjusting for age, BMI, smoking and snus status, cigarette pack-year and investigation season. The controls are the reference group.
